# Antihypertensive medication prescription patterns and time trends for newly-diagnosed uncomplicated hypertension patients in Taiwan

**DOI:** 10.1186/1472-6963-8-133

**Published:** 2008-06-18

**Authors:** Pang-Hsiang Liu, Jung-Der Wang

**Affiliations:** 1 Institute of Occupational Medicine and Industrial Hygiene, College of Public Health, National Taiwan University, Taipei, Taiwan; 2 National Center of Excellence for Clinical Trial and Research, National Taiwan University Hospital, Taipei, Taiwan; 3 Departments of Internal Medicine and Environmental and Occupational Medicine, National Taiwan University Hospital, Taipei, Taiwan

## Abstract

**Background:**

Knowledge of existing prescription patterns in the treatment of newly-diagnosed hypertension can provide useful information for improving clinical practice in this field. The aims of this study are to determine the prescription patterns and time trends for antihypertensive medication in newly-diagnosed cases of uncomplicated hypertension in Taiwan and to compare these with current clinical guidelines.

**Methods:**

A total of 6,536 newly-diagnosed patients with uncomplicated hypertension, aged ≥30 years, were identified from the representative 200,000-person sample in the computerized reimbursement database of the National Health Insurance in Taiwan. These patients were followed from 1998 to 2004 with all diagnoses, prescription data and medication charges being retrieved for subsequent analysis.

**Results:**

Prescription patterns varied by age, gender and clinical facilities, with mono-therapies being found to be dominant in the first year, albeit declining over time. Calcium channel blockers and beta-blockers were the most frequently prescribed antihypertensive drugs, either alone or in combinations. Although least expensive, the prescription rates of diuretics were low, at 8.3% for mono-therapies and 19.9% overall. The prescription rate for angiotensin receptor blockers (ARBs) was elevated considerably over time. After controlling for other related factors by multiple logistic regression analysis, ARBs were found to be prescribed mainly by medical centers or regional hospitals.

**Conclusion:**

These findings indicate the existence of a gap between current clinical practice and the desired goal of cost-effectiveness in antihypertensive treatment in Taiwan, which should be corrected.

## Background

Hypertension, a leading contributor to the global burden of causes of disease, continues its upward growth trend [1-4]. Poor control of this highly prevalent disease can lead to the development of ischemic heart disease, heart failure, stroke and chronic renal insufficiency [3-7]. Along with its comorbidities, hypertensive related conditions have accounted for almost a third of the total causes of death in Taiwan in recent years [8]; in 2003 the total pharmaceutical expenditure on antihypertensive medication was US$0.32 billion, accounting for approximately 27% of the overall annual outpatient pharmaceutical expenditure on western-style medicines [9].

As a result of various clinical trials and studies, a range of clinical guidelines on antihypertensive treatment have been published over the past decade [10-15]. Based on clinical evidence and cost-effectiveness [16-18], guidelines developed by the Joint National Committee (JNC) in the United States [11] and the National Institute for Health and Clinical Excellence (NICE) in the United Kingdom [13] recommended that diuretics (particularly thiazide-type diuretics) should be the drug of first choice for patients with no compelling indications. However, the results of various studies have shown that adherence to such clinical guidelines and recommendations are not at all uniform; indeed, they have been found to vary by time period and country, and by the characteristics of patients and physicians [19-23].

Taiwan's National Health Insurance (NHI) has not yet established a definite guideline for antihypertensive drug therapy. Given the enormous growth in healthcare expenditure within the NHI (from US$13.9 billion in 1997 to US$20.5 billion in 2005) [24] and the limited resources for healthcare, there is a clear need to explore physician practices, including prescription trends, in antihypertensive and other therapies [25,26].

The computerized reimbursement database of the NHI in Taiwan provides us with a valuable opportunity to assess the real practice patterns of antihypertensive pharmaceutical therapies. The NHI program, which is a mandatory nationwide health insurance system, was implemented in Taiwan in March 1995. Overall coverage continues to rise, from 96.2% in 2000 to 98.3% in 2006, and almost the entire population of Taiwan is now covered by the system [27]. Furthermore, in contrast to the NHI systems of many Western nations, patients in Taiwan are free to choose care providers in a competitive healthcare market.

The objectives of this study are to determine antihypertensive medication prescription patterns and time trends among newly-diagnosed cases of uncomplicated hypertension in Taiwan, to attempt to identify the determinants of the choice of first-line drug therapy, and to investigate the pharmaceutical costs associated with different antihypertensive agents.

## Methods

### Study population

This study uses a 200,000-person representative random sample from the computerized reimbursement database of the NHI, between January 1997 and December 2004. Details on the gender and date of birth of the patients, the date of prescription, commercial names of drugs, drug dosages/duration and costs for each prescription are recorded in the reimbursement files.

Patients initially identified were newly-diagnosed with essential hypertension on at least three occasions, were being treated for this condition, and had received their first antihypertensive medication between 1 January 1998 and 31 December 2004. In order to verify that a case was a new one, a period of at least one year was required (January to December of 1997) without any treatment and/or diagnosis relating to hypertension.

To prevent potential confounding by comorbidities in the prescription patterns of antihypertensive agents at different clinical facilities, patients diagnosed with suspected diabetes mellitus, ischemic heart disease, diseases of pulmonary circulation, other forms of heart diseases (including dysrhythmia and heart failure), stroke or renal diseases were excluded from the sample. In order to ensure adherence to these criteria, any of the above diagnoses may not have appeared in any hospitalization file prior to the patient having been diagnosed as hypertensive, and the diagnoses may not have appeared more than three times in ambulatory outpatient files. We discarded those diagnoses appeared only once or twice in ambulatory outpatient files to exclude suspected or uncertain cases where claims were filed to allow for further diagnostic examination.

### Prescription patterns of new cases of hypertension

All antihypertensive drug prescription records from ambulatory care claims and prescriptions dispensed at contracted pharmacies were retrieved and analyzed for our sample of newly-diagnosed patients aged ≥30 years. Patients were stratified by gender and age, with age being split into two sub-groups: the younger group (30–54 years of age) and the older group (≥55 years). The clinical facilities were classified into four types, medical centers, regional hospitals, local hospitals and primary care clinics, based upon the level of medical care provided and the size of the institution as recognized by the NHI.

Antihypertensive drugs were categorized according to the 1999 World Health Organization-International Society Hypertension Guidelines for the Management of Hypertension (WHO/ISH, 1999) and the Seventh Report of the Joint National Committee on Prevention, Detection, Evaluation and Treatment of High Blood Pressure (JNC 7) [10,11]. Six major categories of antihypertensive drugs generally are available, including angiotensin-converting enzyme (ACE) inhibitors, angiotensin receptor blockers (ARBs), beta-blockers, calcium channel blockers (CCBs), diuretics, and others (all other antihypertensive classes including alpha-blockers).

Prescriptions for a chronic disease in Taiwan, such as hypertension, most frequently involved the prescribing of drugs for 28- to 90-day periods, which would allow the patient visit a doctor every one to three months. Since each prescription may have contained different combinations of drugs and durations of medication, analysis of the data was undertaken using the prescription rate as calculated as the number of prescriptions containing a specific antihypertensive agent divided by the total number of prescriptions. A comparison of the prescription time trend was undertaken for each year, beginning with the first antihypertensive prescription. Daily drug costs, excluding all pharmacy service fees or other peripheral costs, were also calculated for each prescription. The drug costs are set by the Bureau of NHI and universally applied to clinical facilities regardless of their sizes.

### Statistical analysis

After being weighted by duration of medication, daily drug costs are expressed as time-weighted means, while other results are expressed as means ± standard deviation (SD). The Chi-square test was carried out to determine the statistical significance of the differences between the prescription rates, with the Cochran-Armitage test also performed to assess the linear time trends over the sample period from the time of the initial treatment. Means of daily drug costs were compared using the Student t-test. Finally, multiple logistic regression analysis was performed to identify possible influential factors as a result of the prescribing of a single class of antihypertensive medication as a mono-therapy. SAS version 9.1 for Windows was used for the analysis of all of the data in this study. All tests were two-sided, and a p-value of <0.05 was considered statistically significant. Whenever multiple comparisons were performed, Bonferroni adjustments were made accordingly.

## Results

The dataset contained a total of 15,835 patients over the age of 30 years who had received their initial dose of antihypertensive drugs for essential hypertension between 1 January 1998 and 31 December 2004. Of this total, 9,299 were excluded on the basis that one or more earlier comorbidities had been recorded. We were therefore left with a total of 6,536 patients and 178,754 prescriptions for antihypertensive agents for subsequent analysis.

Of the total sample of 6,536 patients, 3,268 (50.0%) were women and 49.3% were ≥55 years old, with a mean of 55.9 and SD of 12.3 years. The mean follow-up duration after the first prescription of antihypertensive medication was 42.8 ± 27.2 months, while the average number of overall prescriptions was 27.3 ± 26.0. Each prescription included 1.64 ± 0.84 antihypertensive drugs prescribed for an average period of 22.3 ± 10.5 days. The mean number of actual medical visits over the entire period of study was 25.1 ± 24.5.

### Antihypertensive prescriptions among newly-diagnosed patients

**Figure 1:**
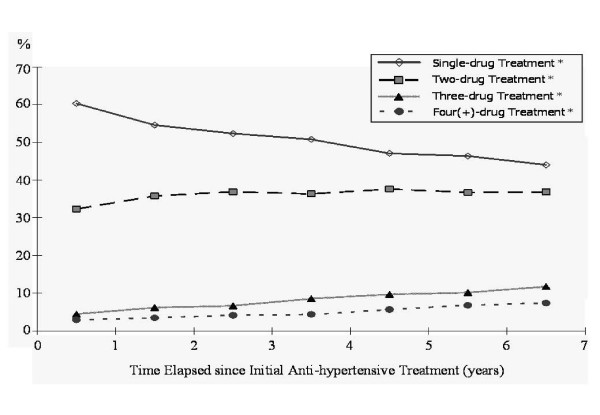
**Prescription pattern time trends for combinations of mono-, two-, three- and four(+)-drug treatment therapies**. Note: * indicates p-value <0.0125 under the Cochran-Armitage trend test, being significant with Bonferroni adjustment for multiple comparisons (p < 0.05/4 = 0.0125).

Over half of the prescriptions for newly-diagnosed cases of uncomplicated hypertension involved single antihypertensive drug therapy (n = 94,797; 53.0%), with women and older patients receiving more mono-therapies. Medical centers and regional hospitals prescribed more combination therapies, as compared with primary care clinics (Table 1). The percentage of mono-therapy treatments declined over time from the initial diagnosis, whereas there was a gradual increase in the percentage of combination therapies (Figure 1). The 10 most frequently prescribed antihypertensive regimens, ranked in order of prescribing frequency, were as follows: CCBs (17.7%), beta-blockers (14.5%), ACEIs (8.2%), CCBs + beta-blockers (7.7%), others (5.3%), diuretics (4.4%), CCBs + ACEIs (4.0%), ARBs (3.0%), CCBs + ARBs (2.6%), beta-blockers + diuretics (2.4%).

**Table 1:** Prescription patterns of antihypertensive therapies for newly-diagnosed uncomplicated hypertension patients, 1998–2004^a^

	Treatment regimen								
		
Variables	Mono-therapy^b^		Two-drug combinations^b^		Three-drug combinations^b^		Four(+)-drug combinations^b^		Total No. of prescriptions^c^
					
	No.	%	No.	%	No.	%	No.	%	
Patient gender									
Male	44 738	51.36^#^	31 494	36.16^#^	6 815	7.82^#^	4 058	4.66^#^	87 105
Female	50 059	54.62	31 927	34.84	6 024	6.57	3 639	3.97	91 649

Patient age (years)									
<55	40 357	50.67^#^	29 784	37.39^#^	6 130	7.70^#^	3 380	4.24	79 651
≥55	54 440	54.93	33 637	33.94	6 709	6.77	4 317	4.36	99 103

Type of clinical facility^d^									
Medical center	16 721	48.76^#^	12 693	37.02	3 897	11.37^#^	978	2.85^#^	34 289
Regional hospital	14 809	49.75^#^	10 453	35.11^#^	3 687	12.39^#^	819	2.75^#^	29 768
Local hospital	18 258	59.03^#^	9 745	31.51^#^	2 395	7.74^#^	531	1.72^#^	30 929
Primary care clinic	44 997	53.73	30 520	36.44	2 860	3.42	5 369	6.41	83 746

Total Nos.	94 797	53.03	63 421	35.48	12 839	7.18	7 697	4.31	178 754

Notes:

^a ^Total sample number = 6 536 patients.

^b ^No. refers to the number of prescriptions under each treatment regimen; % refers to the percentage of the total drugs prescribed under the four treatment regimens.

^c ^As a result of missing data, the sum of the total number of prescriptions over the four types of clinical facilities is smaller than the overall number of prescriptions.

^d ^Pairwise group comparisons are performed taking primary care clinics as the reference.

^# ^Significant p-value with the Bonferroni-adjusted α-level, p < 0.0025

A summary of the total number of prescriptions for the different categories of antihypertensive drugs is provided in Table 2, where it is shown that the most frequently prescribed antihypertensive agents were CCBs (n = 92,574; 51.8%), with beta-blockers as the second most frequently prescribed, followed by ACE inhibitors, diuretics, others and ARBs.

**Table 2:** Distribution of antihypertensive drugs for newly-diagnosed uncomplicated hypertension patients, by gender, age and clinical facility, 1998–2004^a^

	Class of drug^b^												Total No. of prescriptions^b^
		
Variables	Diuretics		Beta-blockers		CCBs^c^		ACE inhibitors^c^		ARBs^c^		Others		
							
	No.	%	No.	%	No.	%	No.	%	No.	%	No.	%	
Patient gender													
Male	15 525	17.82^#^	34 703	39.84^#^	46 468	53.35^#^	22 825	26.20^#^	10 635	12.21^#^	15 212	17.46^#^	87 105
Female	19 981	21.80	40 941	44.67	46 106	50.31	21 306	23.25	10 339	11.28	8 826	9.63	91 649

Patient age (years)													
<55	13 556	17.02^#^	39 445	49.52^#^	40 270	50.56^#^	21 282	26.72^#^	10 421	13.08^#^	7 578	9.5^#^	79 651
≥55	21 950	22.15	36 199	36.53	52 304	52.78	22 849	23.06	10 553	10.65	16 460	16.61	99 103

Type of clinical facility^d^													
Medical center	7 729	22.54^#^	14 463	42.18^#^	18 675	54.46^#^	6 169	17.99^#^	7 764	22.64^#^	3 122	9.10^#^	34 289
Regional hospital	6 025	20.24	12 320	41.39^#^	17 058	57.30^#^	6 170	20.73^#^	5 637	18.94^#^	2 958	9.94^#^	29 768
Local hospital	5 005	16.18^#^	11 303	36.54^#^	17 849	57.71^#^	5 464	17.67^#^	3 594	11.62^#^	3 905	12.63^#^	30 929
Primary care clinic	16 745	19.99	37 540	44.83	38 988	46.56	26 322	31.43	3 966	4.74	14 053	16.78	83 746

Total Nos.	35 506	19.86	75 644	42.32	92 574	51.79	44 131	24.69	20 974	11.73	24 038	13.45	178 754

Notes:

^a ^Total sample number = 6 536 patients.

^b ^No. refers to the number of prescriptions for each class of drug; % refers to the percentage of the total prescriptions for the six classes of drugs. As a result of missing data, the sum of the total number of prescriptions over the four types of clinical facilities is smaller than the overall number of prescriptions. The sum of the prescription rates for all six classes of drugs exceeds 100% because the average prescription contained more than one drug.

^c ^CCBs = calcium channel blockers; ACE = angiotensin-converting enzyme; ARBs = angiotensin receptor blockers.

^d ^Pairwise group comparisons are performed taking primary care clinics as the reference.

^# ^Significant p-value with the Bonferroni-adjusted α-level, p < 0.0017

**Figure 2:**
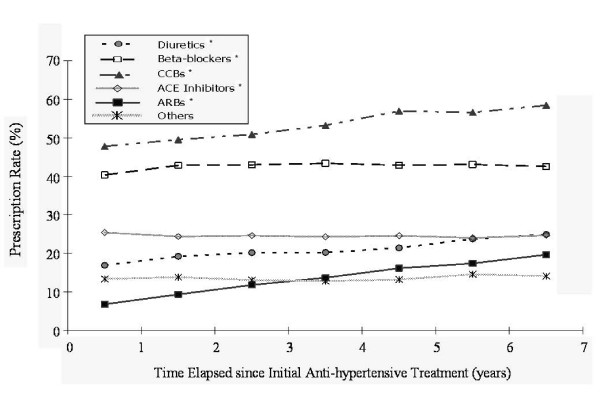
**Prescription distribution time trends for antihypertensive agents**. Note: * indicates p-value <0.0083 under the Cochran-Armitage trend test, being significant with Bonferroni adjustment for multiple comparisons (p < 0.05/6 = 0.0083).

The prescription rate for ARBs, which was the highest in medical centers (22.6%), was almost five times the rate for primary care clinics, and was also higher than the prescription rate for ACE inhibitors and diuretics. There was an increase with time in the number of prescriptions for ARBs, CCBs and diuretics, whereas the number of prescriptions for ACE inhibitors remained stable (Figure 2).

### Mono-therapies for new cases of uncomplicated hypertension

Among all of the mono-therapy prescriptions, the most frequently prescribed antihypertensive agents were CCBs (n = 31,711; 33.5%) and beta-blockers (n = 25,835; 27.3%). Older patients (aged over 55 years) were treated with CCBs more often than younger patients, with beta-blockers being more frequently prescribed among the latter group.

The prescription rates for beta-blockers were higher among women and younger patients (p < 0.0001), while the prescription rates for diuretics were higher among women and older patients (p < 0.0001). In contrast, ACE inhibitors and ARBs were more frequently prescribed for younger patients. Medical centers and regional hospitals were found to have prescribed ARBs much more often than primary care clinics (p < 0.0001), where the prescribing of ACE inhibitors was found to be much more common (p < 0.0001) (Table 3).

**Table 3:** Distribution of mono-therapy antihypertensive drug prescriptions for newly-diagnosed uncomplicated hypertension patients^a^

	Class of drug^b^												Total No. of prescriptions^b^
		
Variables	Diuretics		Beta-blockers		CCBs^c^		ACE inhibitors^c^		ARBs^c^		Others		
							
	No.	%	No.	%	No.	%	No.	%	No.	%	No.	%	
Patient gender													
Male	2 974	6.65^#^	11 602	25.93^#^	14 881	33.26	7 004	15.66	2 561	5.72	5 716	12.78^#^	44 738
Female	4 849	9.69	14 233	28.43	16 830	33.62	7 619	15.22	2 822	5.64	3 706	7.40	50 059

Patient age (years)													
<55	2 366	5.86^#^	13 627	33.77^#^	12 083	29.94^#^	7 134	17.68^#^	2 677	6.63^#^	2 470	6.12^#^	40 357
≥55	5 457	10.02	12 208	22.42	19 628	36.05	7 489	13.76	2 706	4.97	6 952	12.77	54 440

Type of clinical facility^d^													
Medical center	1 067	6.38^#^	4 474	26.76^#^	5 841	34.93^#^	2 255	13.49^#^	2 099	12.55^#^	985	5.89^#^	16 721
Regional hospital	1 026	6.93^#^	4 144	27.98	5 690	38.42^#^	1 710	11.55^#^	1 272	8.59^#^	967	6.53^#^	14 809
Local hospital	1 363	7.47^#^	4 171	22.84^#^	8 186	44.84^#^	1 728	9.46^#^	920	5.04^#^	1 890	10.35^#^	18 258
Primary care clinic	4 367	9.71	13 043	28.99	11 991	26.65	8 927	19.84	1 089	2.42	5 580	12.40	44 997

Total Nos.	7 823	8.25	25 835	27.25	31 711	33.45	14 623	15.43	5 383	5.68	9 422	9.94	94 797

Notes:

^a ^Total sample number of prescriptions = 94 797.

^b ^No. refers to the number of prescriptions for each class of drug; % refers to the percentage of the total prescriptions for the six classes of drugs. As a result of missing data, the sum of the total number of prescriptions over the four types of clinical facilities is smaller than the overall number of prescriptions.

^c ^CCBs = calcium channel blockers; ACE = angiotensin-converting enzyme; ARBs = angiotensin receptor blockers.

^d ^Pairwise group comparisons are performed taking primary care clinics as the reference.

^# ^Significant p-value with the Bonferroni-adjusted α-level, p < 0.0017

**Figure 3:**
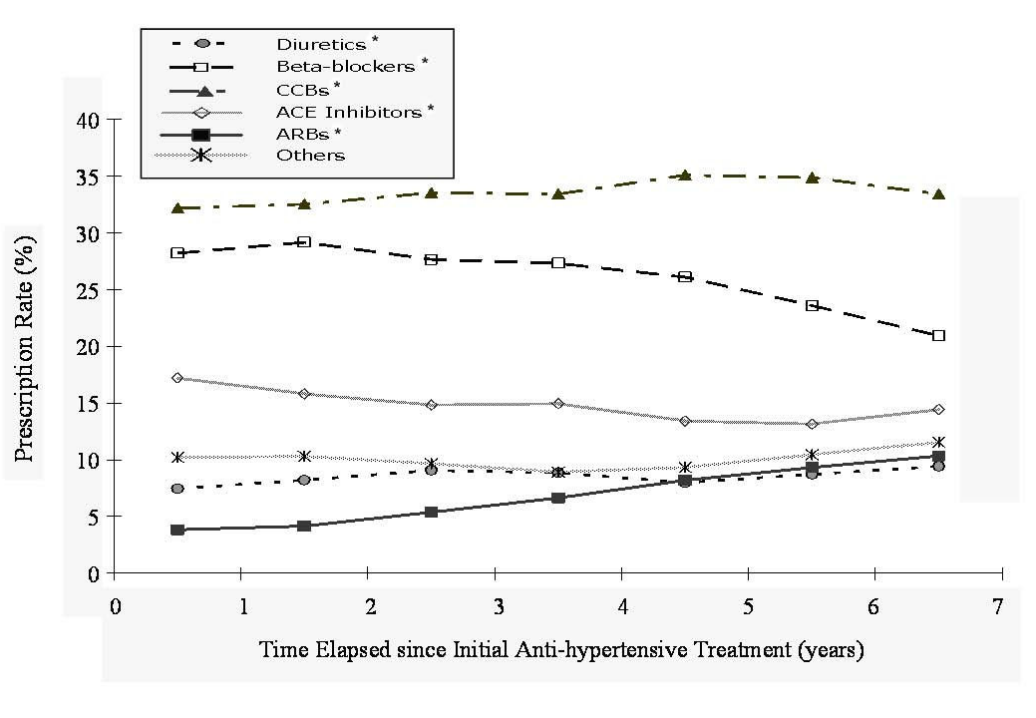
**Time trends for single-drug antihypertensive treatment**. Note: * indicates p-value <0.0083 under the Cochran-Armitage trend test, being significant with Bonferroni adjustment for multiple comparisons (p < 0.05/6 = 0.0083).

With the passage of time from the date of the initial therapy, there was a significant increase in the prescription rate for ARBs, from 3.8% in the first year to 10.3% in the seventh year (p < 0.0001). There was also an increase over time in mono-therapies comprising diuretics; however, there was a reduction over time in the trends for mono-therapies involving beta-blockers or ACE inhibitors (p < 0.0001). The time trends for mono-therapies are summarized in Figure 3.

### Daily drug costs for different antihypertensive mono-therapies

The daily costs for mono-therapy medication, in order from low to high, are as follows. Diuretics were the cheapest with a mean of US$0.17, followed by beta-blockers (US$0.27) and others (US$0.28). The costs for CCBs and ACE inhibitors were almost the same (US$0.56), while the costs for ARBs, at a daily average of US$0.85, were about five times those of diuretics. With the exception of the class of 'other' drugs, the means of the daily drug costs did not vary significantly by gender, age or clinical facility.

### Factors associated with initial ARB mono-therapy prescriptions

As Table 4 shows, following adjustment by multiple logistic regression analysis, those prescriptions involving ARBs as an antihypertensive mono-therapy were found to be associated with subsequent diagnoses of diabetes mellitus (odds ratio (OR) = 1.5; 95% CI: 1.4–1.7), regional hospitals (OR = 3.6, 95% CI: 3.3–3.9), medical centers (OR = 5.8, 95% CI: 5.3–6.2), and the period after the year 2001 (OR = 2.4 for 2001–2, and 4.5 for 2003–2004).

**Table 4:** Multiple logistic regression estimates of ARB and ACE inhibitor mono-therapy prescription characteristics for newly-diagnosed uncomplicated hypertension patients, 1998–2004*

Variables	ARBs		ACE inhibitors	
		
	OR	95% CI	OR	95% CI
Patient gender				
Female (reference)	1.00	-	1.00	-
Male	0.99	0.94–1.05	1.07	1.03–1.11

Patient age (years)				
30–54 (reference)	1.00	-	1.00	-
≥55	0.74	0.70–0.78	0.75	0.72–0.78

Geographical region				
Northwest (reference)	1.00	-	1.00	-
Midwest	0.74	0.68–0.80	0.98	0.93–1.02
Southwest	0.67	0.62–0.72	0.86	0.82–0.90
Eastern	1.34	1.19–1.52	1.39	1.29–1.49
Offshore islands	2.78	2.21–3.49	0.45	0.35–0.57

Type of clinical facility				
Primary care clinics (reference)	1.00	-	1.00	-
Local hospitals	2.17	1.98–2.38	0.43	0.41–0.45
Regional hospitals	3.55	3.26–3.87	0.52	0.49–0.55
Medical centers	5.77	5.32–6.25	0.63	0.60–0.66

Time elapsed since initial therapy				
1 year or less (reference)	1.00	-	1.00	-
2–3 years	1.00	0.92–1.09	0.93	0.89–0.97
4–7 years	1.12	1.03–1.21	0.95	0.90–1.00

Comorbidity after hypertension^†^				
Diabetes mellitus	1.55	1.42–1.68	1.36	1.28–1.44
Ischemic heart disease	1.10	1.02–1.19	0.78	0.73–0.82
Stroke	1.02	0.93–1.11	0.98	0.91–1.05
Chronic renal disease	1.07	0.94–1.22	0.92	0.83–1.02

Calendar years				
1998–2000 (reference)	1.00	-	1.00	-
2001–2002	2.35	2.12–2.62	0.87	0.83–0.91
2003–2004	4.45	4.01–4.94	0.79	0.75–0.83

Notes:

* The odds ratio (OR) for each variable was adjusted for all other variables listed in the table.

^† ^The reference group is subjects with no corresponding comorbidity for each category after hypertension.

## Discussion

This is one of the first studies of its kind to undertake an assessment of the national prescription patterns and time trends in Taiwan for antihypertensive medication for uncomplicated hypertension. We found that whether in mono-therapies or overall treatment, CCBs were the most commonly prescribed drugs, followed by beta-blockers. Amongst all of the mono-therapies, the lowest average daily medication costs were for diuretics, at less than one third of the costs for CCBs or ACE inhibitors, and about one fifth of the costs for ARBs. The prescription rate for diuretics was, however, surprisingly low, accounting for only 8.3% of all mono-therapies, and indeed the diuretic prescription rate was the second lowest of all, only after ARBs (5.7%).

Beginning in 2006–7, the National Health Research Institutes of Taiwan has begun to draft clinical guidelines for various health conditions, including the treatment of hypertension. However, most physicians seemed to accept the recommendation of the US JNC or the WHO/ISH guideline during the time of this study. The evidence-based clinical guidelines for antihypertensive treatment published by both the JNC in 2003 [11] and the NICE in 2004 [13] contained recommendations for low dosages of thiazide diuretics as the first-line drug for essential hypertension with no compelling indications. Such a recommendation also appeared in the 2003 statement published by the WHO/ISH [28]; and indeed, diuretics have been found to be the mainly prescribed class of antihypertensive drugs in the United Kingdom, Denmark and the United States [20,29,30].

Researchers have indicated a substantial potential for cost savings if thiazides are prescribed rather than other more expensive drugs for treatment of hypertension [31,32]. Given that the prescription pattern in Taiwan appears to utilize more non-thiazide medications that are generally more expensive, this issue would be worthy of further study with the aim of comparing the cost-effectiveness of antihypertensive treatment in Taiwan with those of other countries.

Considerable variation in antihypertensive prescribing patterns exists internationally. Fretheim and colleagues compared the sale figures of antihypertensive drugs for six countries, reporting that thiazide diuretics accounted for 25% of consumption in the UK, while the corresponding figure for Norway was only 6% [30]. According to our assessment, thiazide diuretics accounted for 7.2% of overall antihypertensive drugs prescribed for uncomplicated hypertension in Taiwan. The relatively low prescription rate of thiazides for antihypertensive treatment appears similar to those of Norway and France, and is very different from that of the UK or Denmark.

In the absence of any guideline or effective regulations on prescribing behavior for clinicians, the current prescription pattern in Taiwan is probably a reflection of the mixed effect of the preferences of physicians, the hypotensive efficacies of medications, and the tolerance levels of patients. Under a healthcare system of mixed conventional medicine and traditional Chinese medicine, Taiwanese patients appear to dislike diuretics for the treatment of hypertension possibly because of the label 'diuresis', a term generally regarded as treating 'edema' and affecting one's kidney function in traditional Chinese medicine. The mono-therapy prescription rate for diuretics, at less than 10%, albeit with a slightly increasing trend with the passage of time (as shown in Figures 2 and 3), also implies that diuretics are currently considered in Taiwan to be only a second- or third-line medication; thus, there would appear to be considerable room for improvement, in terms of greater adherence to the existent clinical guidelines based on evidence as well as cost-effectiveness [16,18].

The trend toward increasing numbers of prescriptions involving ARBs, as summarized in Table 4, is also worthy of some attention. Indeed, the multiple logistic regression analysis indicated that the calendar year and the size of the clinical facility were actually the major determinants, although we deliberately restricted our subjects to those newly-diagnosed with uncomplicated hypertension and mono-therapies to prevent systematic differences in patients' characteristics. We have also found that primary care clinics prescribed diuretics more frequently than the larger medical facilities. We suspect that differences in cost consciousness may be an important contributor to this particular phenomenon, since the current reimbursement policy within the NHI program seems less restrictive on medical centers and regional hospitals, as compared with primary local clinics. Another possibility is that physicians in large medical facility are more frequently exposed to new drugs and tending to readily accept the latest, or most up-to-date, medications [33]. In fact, the number of ARBs on the market increased from 2 to 9 during the period of 1998–2004. Some studies suggest that promotional activities of pharmaceutical industry have a major impact on physicians' prescribing patterns [34,35]. More evidence needs to be collected to corroborate these beliefs.

One of the limitations of this study is the potential confounding by severity of disease for different levels of clinical facilities. Because the NHI reimbursement database has no link to details on patients' blood pressure levels or laboratory data, we were unable to directly compare the severity of hypertension among various groups of patients. Nonetheless, we have limited our study subjects to newly diagnosed cases of uncomplicated hypertension with mono-therapies, and for them there is no restriction on selection of doctors under the NHI in Taiwan. In such a way, hypertensive patients initially treated at different clinical facilities might not be so much different in severity. Moreover, Table 4 indicated that medical centers used more ARBs and less ACE inhibitors after adjustment for other determinants. As the efficacy of these two types of antihypertensive medication is similar [15], it seems that ARBs might be prescribed to substitute for some ACE inhibitors in medical centers or regional hospitals and this trend probably was unrelated to the different severity of hypertension.

The NHI database provides a 200,000-person sample representing almost 1% of the overall population of 22.9 million people in Taiwan; thus, we estimate that there may have been up to 0.75 million newly-diagnosed cases of uncomplicated hypertension in Taiwan during the seven-year period of this study. If the daily drug costs for uncomplicated hypertension could be reduced by an average of US$0.3–0.6, this would result in annual savings of up to US$82–163 million in overall pharmaceutical expenditure within Taiwan's NHI. If such action were extended to incorporate all prevalent cases of hypertension throughout Taiwan, the total amount of annual savings on costs for antihypertensive drugs could even run to US$0.2 billion. Under the current limited resources, this could clearly make the NHI much more sustainable [26].

## Conclusion

The initial prescription patterns for antihypertensive therapies for uncomplicated hypertension in Taiwan seem to be inconsistent with the current international clinical guidelines. Although diuretics are the least expensive class of antihypertensive drugs, they are nevertheless being used as a second- or third-line method of medication, with a notably low prescription rate. There has been a growing trend in the prescribing of ARBs as the initial choice of therapy for uncomplicated hypertension, particularly in medical centers and regional hospitals. These results indicate a need for greater awareness of the evidence-based guidelines for antihypertensive drug therapy amongst physicians and the general public.

Given the existence of the national health insurance system in Taiwan, there is still significant room for improvement in the cost-effectiveness of antihypertensive treatment [16,25]. We recommend reaching a consensus on this matter and developing a domestic clinical guideline taking cost-effectiveness into consideration as soon as possible.

## Competing interests

The authors confirm that they have no interests which might be perceived as giving rise to any form of bias or conflict of interest.

## Authors' contributions

Both authors formulated the research question and design of the study. P–HL carried out the study and J–DW advised on the analyses. P–HL drafted the manuscript, and both authors critically revised it. Both authors read and approved the final manuscript.

## Pre-publication history

The pre-publication history for this paper can be accessed here:


